# Forced convection flow of water conveying AA7072 and AA7075 alloys-nanomaterials on variable thickness object experiencing Dufour and Soret effects

**DOI:** 10.1038/s41598-022-10901-w

**Published:** 2022-04-28

**Authors:** Umair Khan, A. Zaib, Sakhinah Abu Bakar, Anuar Ishak

**Affiliations:** 1 Department of Mathematical Sciences, Faculty of Science and Technology, Universiti Kebangsaan Malaysia, UKM, 43600 Bangi, Selangor Malaysia; 2 Department of Mathematics and Social Sciences, Sukkur IBA University, Sukkur, 65200 Sindh Pakistan; 3 Department of Mathematical Sciences, Federal Urdu University of Arts, Science and Technology, Gulshan-e-Iqbal Karachi, 75300 Pakistan

**Keywords:** Applied mathematics, Computational science

## Abstract

Hybrid nanofluids containing titanium alloy particles have a large class of applications in industrial plastics and soaps, microsensors, aerospace material designs, optical filters, nanowires, surgical implants, and a variety of biological applications. This paper presents a mathematical analysis of Soret and Dufour impacts on the radiative flow through a thin moving needle of binary hybrid alloys nanoparticles. The transformed ordinary differential equations are solved numerically using the built-in function, bvp4c, in MATLAB software. The influences of all relevant parameters are shown in figures and tables. Two outcomes are developed for a precise range of the velocity ratio parameter. In particular, dual solutions are obtained when the needle and the fluid move in the opposite directions, while the solution is unique when they move in the same direction. The outcomes disclose that addition of nanoparticles into the base fluid upsurges the shear stress and the Nusselt number while decreasing the Sherwood number. Meanwhile, an upsurge in the needle size results in an uplift of the temperature and the concentration for the upper branch solution, whereas the velocity declines.

## Introduction

Due to the high need for heat transfer in industrial and technical applications, researchers and scientists have been working hard for several years to develop new methods to boost the rate of heat transmission. As a result, the issue of improving heat transmission in a variety of industrial equipment has seen an inventive push for a more detailed investigation of heat capacities. They are widely employed in a variety of engineering applications, including heat exchangers, nuclear reactors, aircraft technology, microelectronics, transportation, and biomedicine, among others. By combining the number of nanoparticles with normal liquids such as water, ethylene glycol, and oil, a new category of liquids known as nanofluid with improved heat transport rate has been created. The term nanofluid refers to a single type of nanoparticles suspension in a regular liquid. Choi and Eastman^1^ were the first to come up with the concept of a nanofluid, which consists of nanomaterials scattered in a regular fluid. Sheikholeslami et al.^2^ inspected the magnetic field between warmth cylinders containing water-based Al_2_O_3_ particles with an icy square. Sheremet et al.^3^ inspected the unsteady flow conveying nanoparticles in a cavity with magnetic influence. The activation energy with entropy generation on magnetic fluid flow with nanofluid over a wedge was examined by Zaib et al.^4^ in the presence of a modified Arrhenius function (MAF). Seth et al.^5^ dissected the impression of the magnetic influence on the fluid flow of CNT nanotube past a stretchable sheet in a rotating frame with entropy generation. Nayak et al.^6^ inspected the control of erratic Lorentz forces on 3D flow induced by nano liquid from a stretchable sheet with a radiative effect. Khan et al.^7^ recently inspected the Falkner-Skan flow of Sutterby nanoliquid over a wedge with Cattaneo-Christov flux. An erratic mathematical model leading by subjectively oblique-slip flow in carbon nanotubes suspended nanofluid, aggravated by the magnetic and radiation impact was numerically simulated by Mandal et al.^8^. Nandi et al.^9^ discussed the impact of Arrhenius energy and Hall current on the Williamson nanoparticles 3D flow over a slander stretched sheet with MHD and slip effect. Nandi et al.^10^ utilized a statistical as well as numerical approaches to investigate the heat generation impact on the magnetized flow through a stagnation-point in a nanofluid past a heated stretching sheet with slip effect and activation energy. Recently, Wang et al.^11^ inspected the micropolar time-dependent flow past a curved stretchable sheet in a nano liquid subject to a chemical reaction. They observed that the nano liquid velocity uplift due to curvature parameter. The impact of slip effect on the time-dependent 3D flow through a Maxwell nanofluid induced by gyrotatic microoragnims past an expanding exponential sheet with convective condition was reviewed by Wang et al.^12^. Wang et al.^13^ scrutinized the dipole magnetic consequence on the bioconvection flow of Casson nano liquid from a stretchable cylinder.

Hybrid nanofluid has been created by upgrading the normal nanofluids that can effectively enhance the thermal features utilized in the industrial processes. This novel kind of fluid agent has magnetized the curiosity of several researchers due to its capability in growing and enlightening the features of thermal properties in practical applications like heat exchangers, a system of air-conditioning, automobile industry, biomedical, etc. Therefore, Turcu et al.^14^ and Jana et al.^15^ appeared among the first scholars to use binary hybrid nanoparticles in their practical or experimental investigation. Devi and Devi^16,17^ looked at the mathematical analysis of liquid combined with copper–aluminum nano liquid flow over a stretched sheet with heat transport. The features of heat transfer and fluid flow containing hybrid nanoliquid via a porous erratic shrinking/stretching surface were studied by Waini et al.^18^. Xiong et al.^19^ inspected the slip effect on the 2D flow induced by hybrid nanoliquid past a sheet with Darcy-Forchheimer and entropy generation. Lately, Khan et al.^20^ scrutinized the SPF via shrinking or stretching sheets inducted by micro rotation effects with hybrid nanofluid and presented double solutions.

In the last few years, the problem involving heat transfer and mass transfer induced by nanofluid has multiplied a substantial interest among scholars and researchers due to vital applications in the construction of nuclear reactors, electronic devices, and compact heat exchangers. The Dufour (Du) and Soret (Sr) consequences are well known for their importance in the intermediate double system-induced via molecular weight gases that are most frequently come across in the chemical engineering process for the coupled rate of mass and heat transfer. While species are put on the ground area in a spectrum of fluid with a smaller density than the nearby fluid, the Dufour and Soret impressions become substantial. In addition, the impact of Sr and Du effects can be seen in the different regions of combustion flames, reactor safety, conservation of building energy, and solar collector. For an instant, Sheremet^21^ investigated the impressions of Sr and Du on the features of mass and heat transfer through a square enclosure via a fixed heat-conductive walls. The Dufour and Soret effects on time-dependent flow via a stretched sheet with magnetic field and Hall current were investigated by Zaib and Shafie^22^. Bhattacharyya et al.^23^ looked at the impacts of Du and Sr on stagnation point flow with convective transfer through a shrinking sheet. They presented double solutions for concentration as well as for temperature fields. Ashraf et al.^24^ surveyed the mixed convection flow of a non-Newtonian fluid with a heated stretchable sheet with Dufour and Soret effects. The unsteady slip flow comprised Casson fluid via a nonlinear stretching sheet induced by the consequences of mixed convection, Dufour and Soret effects were highlighted by Ullah et al.^25^. Seth et al.^26^ assesed the 2D natural convective flow provoked by non-Newtonian nanofluid past a linear stretachable sheet with Dufour and Soret impacts. They observed that the wall velocity gradient was enhanced by magnetic field, viscoelasticity, and buoyancy nanoparticle parameter, while thermal buoyancy force has the conflicting effect. Imtiaz et al.^27^ focused on the viscous fluid flow from a stretching curved in the presence of Joule heating, Dufour and Soret effects. Das et al.^28^ examined the Dufour and Soret effects on the magnetohydrodynamic flow of a nano liquid provoked by tangent hyperbolic fluid through a bi-directional stretchable sheet with slip effects. They observed that the Dufour number reduced the temperature of the fluid near the boundary layer, while the Soret number raised the concentration within the boundary layer. Recently, Singha et al.^29^ inspected the Du and Sr effects on the hydromagnetic flow of a water-based nano liquid through an expanding exponential sheet in a non-Darcy medium.

The research on the exchange of heat fluid flow around diverse objects has attracted the interest of researchers because of their significant practical uses, such as wind and airflow, past an aero-plane, etc. Lee^30^ investigated the boundary-layer flow about a horizontally positioned needle by employing viscous incompressible fluid. Narain and Uberoi^31,32^ modified the Lee^30^ work by considering the forced and free convective flow through a placed vertically thin needle (TN). Sulochana et al.^33^ examined the 2D magnetohydrodynamic ferrofluid flow via a moving horizontal needle subjected to viscous dissipative and erratic heat source/sink. The impact of Joule heating on the flow-induced by nanomaterials from a needle was inspected by Upreti and Kumar^34^. Soid et al.^35^ considered the nanoparticle’s flow on a moving needle, where the existence of dual results was reported. Khan et al.^36^ discussed the erratic radiative flow of a hybrid nanomaterials past a TN subject to Lorentz force and activation energy. Waini et al.^37^ calculated the steady flow past a moving porous medium of a thin needle with heat transfer comprising hybrid nanofluid and double solutions. Khan et al.^38^ surveyed the variation of erratic radiative impact on the non-Newtonian fluid flow through a thin needle conveying alloy particles.

A closer examination of the literature on the previously mentioned topics revealed some shortcomings and gaps. To the best of the authors knowledge, no previous studies have investigated the radiative hybrid alloys nanoparticles flow past a thin moving needle with significant impacts of Dufour and Soret included in their research framework. Therefore, inspired by the aforementioned gap of knowledge, our ultimate purpose of the current exploration is to present a mathematical analysis of Dufour and Soret impacts past a thin needle on radiative flow in hybrid alloy nanoparticles (AA7072 and AA7075). The MATLAB built-in function, namely bvp4c is utilized to find double solutions. This critical contribution may aid in the improvement of industrialized production, particularly in the process and manufacturing sectors.

## Mathematical framework of the flow problem

**Figure 1:**
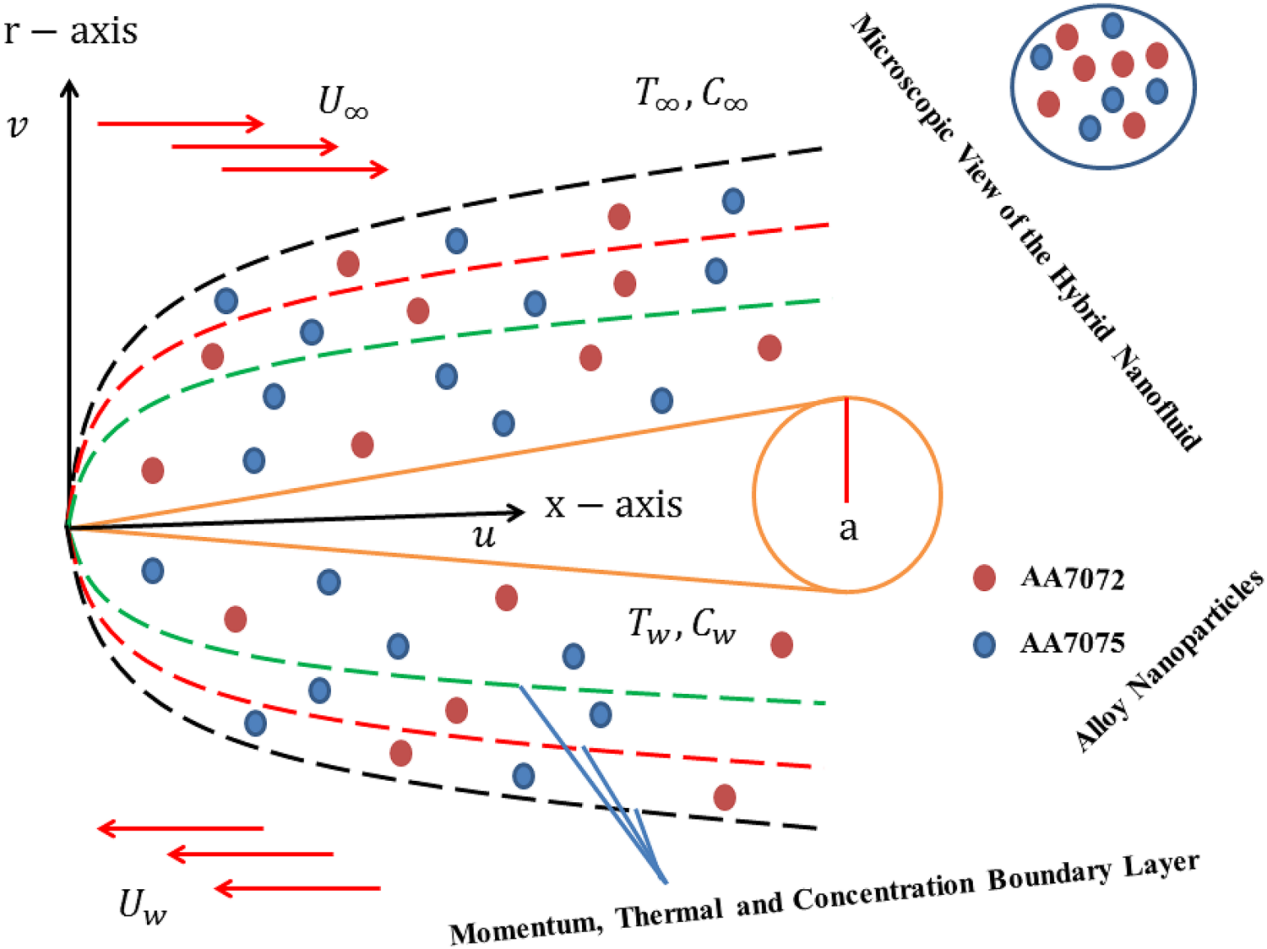
Physical model.

Let us consider the boundary layer flow of experiencing the Soret and Dufour impacts on the forced convective flow of a hybrid nanofluid towards a moving TN considering the radiation effect, as shown in Fig. 1. Here, $\left( {x,r} \right)$ are the requisite posited Cartesian cylindrical coordinates with the $x$-axis considered along the needle and $r$-axis perpendicular to it. The flow is at $r \ge 0$, where $r = \sqrt {\frac{{a\nu_{bf} x}}{U}} = R\left( x \right)$ represents the needle radius. The two dissimilar hybridized (AA7072 and AA7075) alloy nanoparticles and the regular base fluid (water) are mixed to form a new class such as binary hybrid nanofluid. The properties of these hybridized alloy nanomaterials and the regular base fluid are considered constant. Further, it is assumed that the constant velocity of the moving needle is indicated by $U_{w}$ in the direction of contrary or same to the far-field flow of constant speed $U_{\infty }$ with the applicable of composite speed $U = U_{w} + U_{\infty }$ and $q_{r}$ represents the radiative heat flux. Besides, $T_{w}$ and $T_{\infty }$ are signified as the constant surface temperature and the ambient temperature of the base fluid, respectively such that $T_{w} > T_{\infty }$ but $C_{w}$ and $C_{\infty }$ correspond to the respective constant surface concentration and the ambient concentration of the regular (viscous) fluid.

Using the above assumptions and standard boundary-layer scaling or approximations, the posited governing PDEs are [see Devi and Devi^16^ and Salleh et al.^39^]

1 $$ \frac{\partial }{\partial x}\left( {ur} \right) + \frac{\partial }{\partial r}\left( {vr} \right) = 0, $$

2 $$ \rho_{hnf} \left( {u\frac{\partial u}{{\partial x}} + v\frac{\partial u}{{\partial r}}} \right) = \mu_{hnf} \frac{1}{r}\frac{\partial }{\partial r}\left( {r\frac{\partial u}{{\partial r}}} \right), $$

3 $$ u\frac{\partial T}{{\partial x}} + v\frac{\partial T}{{\partial r}} = \frac{{k_{hnf} }}{{\left( {\rho c_{p} } \right)_{hnf} }}\frac{1}{r}\frac{\partial }{\partial r}\left( {r\frac{\partial T}{{\partial r}}} \right) + \frac{{D_{m} K_{T} }}{{c_{p} c_{s} }}\frac{1}{r}\frac{\partial }{\partial r}\left( {r\frac{\partial C}{{\partial r}}} \right) - \frac{1}{{\left( {\rho c_{p} } \right)_{hnf} }}\frac{1}{r}\frac{\partial }{\partial r}\left( {rq_{r} } \right), $$

4 $$ u\frac{\partial C}{{\partial x}} + v\frac{\partial C}{{\partial r}} = D_{B} \frac{1}{r}\frac{\partial }{\partial r}\left( {r\frac{\partial C}{{\partial r}}} \right) + \frac{{D_{m} K_{T} }}{{T_{m} }}\frac{1}{r}\frac{\partial }{\partial r}\left( {r\frac{\partial T}{{\partial r}}} \right). $$

with boundary conditions (BCs) take place as:

5 $$ \left\{ \begin{gathered} T = T_{w} ,\,\,u = U_{w} ,\,\,C = C_{w} ,\,\,v = 0{\text{, at }}r = R(x), \hfill \\ T \to T_{\infty } ,\,\,u \to U_{\infty } {,}\,\,C \to C_{\infty } {\text{ as }}\,r \to \infty . \hfill \\ \end{gathered} \right. $$

where $v$ and $u$ are the given velocity relative components measured in the respective $r -$ and $x -$ axes, $C$ the concentration and $T$ the temperature. Moreover, $D_{B}$ corresponds the Brownian diffusion coefficient, $D_{m}$ is the mass diffusivity coefficient, $c_{p}$ is the heat capacity at uniform pressure, $c_{s}$ is the nanoparticle concentration susceptibility, $K_{T}$ is the ratio of thermal diffusion and $T_{m}$ is the fluid mean temperature. The rest of the mathematical symbols used for the hybrid nanofluid will be defined later in the same section.

The well-established Rosseland approximation which is applicable and describe the radiative heat transfer $q_{r}$ in the limit of an optically thick fluid (hybrid nanofluid) is employed. The related term involves in the aforesaid Eq. (3) is the $q_{r}$ which is articulated as follow (see Bataller^40^; Magyari and Pantokratoras^41^):

6 $$ q_{r} = - \frac{{4\sigma_{s} }}{{3k_{s} }}\frac{{\partial T^{4} }}{\partial r} $$

where $k_{s}$ and $\sigma_{s}$ indicate the respective mean absorption coefficient and the Stefan-Boltzmann constant. Now employing the proper Taylor series for the suggested term $T^{4}$ about a specific point $T_{\infty }$ and leaving the second, third or higher-order power terms, yields $T^{4} \approx 4TT_{\infty }^{3} - 3T_{\infty }^{4}$. Using this simplified expression in Eq. (3), one gets

7 $$ u\frac{\partial T}{{\partial x}} + v\frac{\partial T}{{\partial r}} = \frac{{k_{bf} }}{{\left( {\rho c_{p} } \right)_{hnf} }}\left( {\frac{{k_{hnf} }}{{k_{bf} }} + \frac{{16\sigma_{s} T_{\infty }^{3} }}{{3k_{s} k_{bf} }}} \right)\frac{1}{r}\frac{\partial }{\partial r}\left( {r\frac{\partial T}{{\partial r}}} \right) + \frac{{D_{m} K_{T} }}{{c_{p} c_{s} }}\frac{1}{r}\frac{\partial }{\partial r}\left( {r\frac{\partial C}{{\partial r}}} \right). $$

In addition, the absolute viscosity,$\mu_{hnf}$, the thermal conductivity, $k_{hnf}$, the density, $\rho_{hnf}$ and the specific heat capacity $\left( {\rho c_{p} } \right)_{hnf}$ of the hybrid nanoliquid can be read as follows (see Takabi and Salehi^39^):

8 $$ \left\{ \begin{gathered} \frac{{\mu_{hnf} }}{{\mu_{bf} }} = \frac{1}{{\left( {1 - \phi_{{s_{1} }} - \phi_{{s_{2} }} } \right)^{2.5} }}\,, \, \frac{{\rho_{hnf} }}{{\rho_{bf} }} = \phi_{{s_{1} }} \left( {\frac{{\rho_{{s_{1} }} }}{{\rho_{bf} }}} \right) + \phi_{{s_{2} }} \left( {\frac{{\rho_{{s_{2} }} }}{{\rho_{bf} }}} \right) + \left( {1 - \phi_{{s_{1} }} - \phi_{{s_{2} }} } \right), \hfill \\ \alpha_{hnf} = \frac{{k_{hnf} }}{{\left( {\rho c_{p} } \right)_{hnf} }},\, \, \frac{{\left( {\rho c_{p} } \right)_{hnf} }}{{\left( {\rho c_{p} } \right)_{bf} }} = \phi_{{s_{1} }} \left( {\frac{{\left( {\rho c_{p} } \right)_{{s_{1} }} }}{{\left( {\rho c_{p} } \right)_{bf} }}} \right) + \phi_{{s_{2} }} \left( {\frac{{\left( {\rho c_{p} } \right)_{{s_{2} }} }}{{\left( {\rho c_{p} } \right)_{bf} }}} \right) + \left( {1 - \phi_{{s_{1} }} - \phi_{{s_{2} }} } \right), \hfill \\ \frac{{k_{hnf} }}{{k_{bf} }} = \frac{{\left( {D_{a} + D_{b} } \right)}}{{\left( {D_{a} + D_{c} } \right)}},\,\,\,\,\,\,\,\, \hfill \\ {\text{where}}\,\,D_{a} = \frac{{\left( {\phi_{{s_{1} }} k_{{s_{1} }} + \phi_{{s_{2} }} k_{{s_{2} }} } \right)}}{{\phi_{{s_{1} }} + \phi_{{s_{2} }} }},\left\{ \begin{gathered} D_{b} = 2k_{bf} + 2\left( {\phi_{{s_{1} }} k_{{s_{1} }} + \phi_{{s_{2} }} k_{{s_{2} }} } \right) - 2\left( {\phi_{{s_{1} }} + \phi_{{s_{2} }} } \right)k_{bf} ,\, \hfill \\ D_{c} = 2k_{bf} - \left( {\phi_{{s_{1} }} k_{{s_{1} }} + \phi_{{s_{2} }} k_{{s_{2} }} } \right) + \left( {\phi_{{s_{1} }} + \phi_{{s_{2} }} } \right)k_{bf} , \hfill \\ \end{gathered} \right\} \hfill \\ \end{gathered} \right.. $$

Here $\phi$ is the solid nanoparticle volume fraction ($\phi = 0$ indicates the common pure base fluid, $\phi_{{s_{1} }}$ indicates AA7072 and $\phi_{{s_{2} }}$ indicates AA7075), $\rho_{bf}$, $\rho_{{s_{1} }}$ and $\rho_{{s_{2} }}$ are densities for the common base fluid. Further, $k_{bf}$, $k_{{s_{1} }}$ and $k_{{s_{2} }}$ respectively represent the thermal conductivities of the common base fluid and the two distinct hybridized alloy nanomaterials, $\mu_{bf}$, $\mu_{{s_{1} }}$ and $\mu_{{s_{2} }}$ are absolute viscosities of the common base fluid and the two distinct hybridized alloy nanomaterials and $\left( {\rho c_{p} } \right)_{bf}$, $\left( {\rho c_{p} } \right)_{{s_{1} }}$and $\left( {\rho c_{p} } \right)_{{s_{2} }}$ are the particular heat capacities of the pure base liquid and the two distinct hybridized alloy nanomaterials, respectively. The characteristics of the physical data are shown in Table 1 for the two dissimilar nanoparticles AA7072 and AA7075 along with the common base fluid, water.

**Table 1:** Experimental physical data of water and hybrid nanofluid.

Thermophysical characteristics	H_2_O	AA7075	AA7072
$k \, \left( {\text{W/mK}} \right)$	0.6129	173	222
$\rho \, \left( {{\text{kg/m}}^{{3}} } \right)$	997.1	2810	2720
$c_{p} \, \left( {\text{J/kg K}} \right)$	4179	960	893
$\Pr$	6.2		

Now, the following self-similarity variables (see Khan et al.^36^) are considered:

9 $$ \psi = \nu_{bf} xF\left( \xi \right)\,,\,\,S\left( \xi \right) = \frac{{C - C_{\infty } }}{{C_{w} - C_{\infty } }},\xi = \frac{{Ur^{2} }}{{\nu_{bf} x}},\theta \left( \xi \right) = \frac{{T - T_{\infty } }}{{T_{w} - T_{\infty } }}, $$

where $\nu_{bf}$ is the kinematic viscosity and $\psi$ is the stream function which is demarcated as $ru = \frac{\partial \psi }{{\partial r}}$ and $vr = - \frac{\partial \psi }{{\partial x}}$. Hence, we have

10 $$ u = 2UF^{\prime}\left( \xi \right)\,\,{\text{and}}\,\,v = - \frac{{\nu_{bf} }}{r}\left( {F\left( \xi \right) - \xi F^{\prime}\left( \xi \right)} \right). $$

In addition, by putting $\xi = a$ in Eq. (9), which designates the shape or object and proper size of the variable thickness, $r = R(x)$, the appropriate surface may be represented as $R(x) = \sqrt {\frac{{a\nu_{bf} x}}{U}}$.

Substituting Eq. (9) into the governing Eqs. (2), (4) and (7), the following reduced form of ordinary (similarity) differential equations are obtained:

11 $$ \frac{{\mu_{hnf} /\mu_{bf} }}{{\rho_{hnf} /\rho_{bf} }}2\left( {\xi F^{\prime\prime\prime} + F^{\prime\prime}} \right) + FF^{\prime\prime} = 0, $$

12 $$ \frac{2}{{\Pr \left( {\rho c_{p} } \right)_{hnf} /\left( {\rho c_{p} } \right)_{bf} }}\left( {\frac{{k_{hnf} }}{{k_{bf} }} + \frac{4}{3}R_{d} } \right)\left( {\xi \theta ^{\prime\prime} + \theta ^{\prime}} \right) + F\theta ^{\prime} + 2Du\left( {\xi S^{\prime\prime} + S^{\prime}} \right) = 0, $$

13 $$ \frac{2}{Le}\left( {\xi S^{\prime\prime} + S^{\prime}} \right) + FS^{\prime} + 2Sr\left( {\xi \theta ^{\prime\prime} + \theta ^{\prime}} \right) = 0, $$

subject to BCs

14 $$ \begin{gathered} \theta (a) = 1,\,\,S(a) = 1,\,\,F^{\prime}(a) = \frac{\lambda }{2},\,\,F(a) = \frac{\lambda }{2}a{, }\,\,\, \hfill \\ \theta (\xi ) \to 0{, }F^{\prime}\left( \xi \right) \to \frac{1 - \lambda }{2}, \, S(\xi ) \to 0{\text{ as }}\xi \to \infty , \hfill \\ \end{gathered} $$

where $a$ represents the needle size. Further, $R_{d}$ the radiation parameter, $Le$ the Lewis number, $\Pr$ the Prandtl number, $Du$ the Dufour factor, $Sr$ the Soret factor, and $\lambda$ the velocity ratio parameter, which are expressed as:

15 $$ \begin{gathered} R_{d} = \frac{{4\sigma_{s} T_{\infty }^{3} }}{{k_{s} k_{bf} }},\,\,\lambda = \frac{{U_{w} }}{U},\,\,\Pr = \frac{{\nu_{bf} }}{{\alpha_{bf} }},\,\,Du = \frac{{D_{m} K_{T} \left( {C_{w} - C_{\infty } } \right)}}{{\nu_{bf} c_{p} c_{s} \left( {T_{w} - T_{\infty } } \right)}},\,\,Le = \frac{{\nu_{bf} }}{{D_{B} }}, \hfill \\ Sr = \frac{{D_{m} K_{T} \left( {T_{w} - T_{\infty } } \right)}}{{\nu_{bf} T_{m} \left( {C_{w} - C_{\infty } } \right)}}, \hfill \\ \end{gathered} $$

with $\lambda > 0$ represents the moving needle in alike track as the free-stream flow, $\lambda < 0$ represents the needle against the free-stream flow, and $\lambda = 0$ for a stationary needle.

The physical engineering quantities of interest or gradients are the shear stress $C_{f}$, the local Nusselt number $Nu_{x}$, and the local Sherwood number $Sh_{x}$ which are described as:

16 $$ \begin{gathered} \left( {{\text{Re}}_{x} } \right)^{\frac{1}{2}} C_{f} = \frac{{\mu_{hnf} \left( {\frac{\partial u}{{\partial r}}} \right)_{r = R(x)} }}{{\rho_{bf} U^{2} }} = 4\frac{{\mu_{hnf} }}{{\mu_{bf} }}\sqrt a F^{\prime\prime}\left( a \right), \hfill \\ \left( {{\text{Re}}_{x} } \right)^{{ - \frac{1}{2}}} Nu_{x} = \frac{x}{{k_{bf} \left( {T_{w} - T_{\infty } } \right)}}\left. {\left( { - k_{hnf} \frac{\partial T}{{\partial r}} + \left( {q_{r} } \right)} \right)} \right|_{r = R(x)} = - 2\sqrt a \left( {\frac{{k_{hnf} }}{{k_{bf} }} + \frac{4}{3}R_{d} } \right)\theta ^{\prime}\left( a \right), \hfill \\ \left( {{\text{Re}}_{x} } \right)^{{ - \frac{1}{2}}} Sh_{x} = \frac{x}{{D_{B} \left( {C_{w} - C_{\infty } } \right)}}\left. {\left( { - D_{B} \frac{\partial C}{{\partial r}}} \right)} \right|_{r = R(x)} = - 2\sqrt a S^{\prime}\left( a \right). \hfill \\ \end{gathered} $$

where ${\text{Re}}_{x} = xU/\nu_{bf}$ signifies the Reynolds number.

## Analysis of results and discussion

The non-linear coupled ODEs (11) to (13) along with the constraints (14) are investigated numerically with the help of the bvp4c package in MATLAB software. For numerical computations, the ranges of the controlling parameters are considered as $0.02 < \phi_{1} < 0.04$, $0.02 < \phi_{2} < 0.04$, $0.0 \le R_{d} \le 5.0$, $0.01 \le Du \le 1.0$, $0.001 \le a \le 0.16$, $0.01 \le Sr \le 1.0$, $- 1.0 \le \lambda \le - 6.0$, $0.01 \le Le \le 1.0$ and $\Pr = 6.2$. The thermo-physical data of the ordinary base fluid (water) and the two different types of hybrid alloy nanoparticles (AA7072 and AA7075) are given in Table 1. The acquired outcomes of the reduced drag force for several values of the needle thickness $a$ when $Sr = 0$, $Le = 0$, $Du = 0$, $\lambda = 0$, $R_{d} = 0$, $\phi_{1} = 0$ and $\phi_{2} = 0$ are authenticated with the existing works of Khan et al.^36^, Soid et al.^43^, Salleh et al.^44^, Ishak et al.^45^ and Waini et al.^46^, which are presented in Table 2. The constructed table displays that the outcomes assessment data are in an excellent match with the available information of published works, which support the validity of the current numerical outcomes. Table 3 is organized to illustrate the influences of the various constraints such as the hybrid nanoparticles $\phi_{1}$ and $\phi_{2}$, moving ratio parameter $\lambda$, and the needle size $a$ on the friction factor for both branches (UB and LB) outcomes. The outcomes divulge that the generated values of the friction factor enhance in both solution branches owing to the impact of $\lambda$, $\phi_{1}$ and $\phi_{2}$, while reduces in the respective LB solution due to the sophisticated values of $a$. In addition, the discrepancy of the heat transfer rate for sundry values of $\phi_{1}$ and $\phi_{2}$, the radiation parameter $R_{d}$ and the Dufour number $Du$ for both distinct solution branches are depicted in Table 4. From this table, outcomes are portrayed to see mainly the heat transfer rate which is significantly boosted for the UB as well as the LB outcomes owing to the superior values of $\phi_{1}$, $\phi_{2}$, $R_{d}$ and $Du$. Moreover, the heat transfer rate is slowly augmented in the LB solutions as compared to the UB solutions. Finally, Table 5 highlights the computational values of the local Sherwood number for the UB and LB results in response of the various values of $Le$, $\phi_{1}$, $\phi_{2}$ and $Sr$. The local Sherwood number is augmenting in both branches of the solution while the same pattern is seen in the UB and the opposite pattern is followed for the LB with the hybrid nanoparticles and the Lewis number.

**Table 2:** Comparison of $\left( {{\text{Re}}_{x} } \right)^{\frac{1}{2}} C_{f}$ for several values of needle size $a$ when $\Pr = 1$ and the rest of all comprising parameters are absent.

$a$	Khan et al.^36^	Soid et al.^43^	Salleh et al.^44^	Ishak et al.^45^	Waini et al.^46^	Current Outcomes
0.01	8.4921	8.491454	8.4924453	8.4924	8.491455	8.4920683
0.1	1.2889	1.288778	1.2888300	1.2888	1.288778	1.2888757
0.15	0.9385	–	0.9383388	–	–	0.9384789
0.2	0.7517	0.751665	0.7515725	–	0.751665	0.7516956

**Table 3:** The computational output values of drag force for several values of the mentioned parameters.

$\phi_{1} ,\phi_{2}$	$\lambda$	$a$	$4\sqrt a \frac{{\mu_{hnf} }}{{\mu_{bf} }}F^{\prime\prime}(a)$	
			Upper branch solution	Lower branch solution
0.022	− 3.5	0.1	10.744515	4.7602135
0.026			11.001564	4.8330030
0.030			11.267234	4.9069176
0.034			11.541805	4.9820315
	− 2.0		8.6698889	1.8156426
	− 2.5		9.9338992	2.6608255
	− 3.0		10.944255	3.6943391
	− 3.5		11.611872	5.0010056
		0.070	14.813114	4.8768293
		0.085	13.094559	4.8928971
		0.095	12.087828	4.9543689
		0.1	11.611872	5.0010056

**Table 4:** The computational output values of the local heat flux for sundry values of the mentioned parameters.

$\phi_{1} ,\phi_{2}$	$R_{d}$	$Du$	$- 2\sqrt c \left( {\frac{{k_{hnf} }}{{k_{bf} }} + \frac{4}{3}R_{d} } \right)\theta ^{\prime}(a)$	
			Upper branch solution	Lower branch solution
0.022	2.0	0.4	4.4442242	1.9299000
0.026			4.4927471	1.9325963
0.030			4.5420482	1.9345058
0.034			4.5921239	1.9356261
	1.0		2.5715016	0.9095888
	1.5		3.5824305	1.3683308
	2.0		4.6047635	1.9357822
	2.5		5.6261206	2.5854656
		0.1	4.1057667	1.1847094
		0.3	4.4311727	1.6870532
		0.5	4.7860747	2.1823139
		0.7	5.1731712	2.6670956

**Table 5:** The numerical values of the local mass flux for various controlling parameters.

$\phi_{1} ,\phi_{2}$	$Le$	$Sr$	$- 2\sqrt a S^{\prime}(a)$	
			Upper branch solution	Lower branch solution
0.022	1.0	0.4	1.2385102	0.5949004
0.026			1.2386749	0.5869906
0.030			1.2388286	0.5788070
0.034			1.2389674	0.5703574
	0.07		1.2450814	1.1160845
	0.085		1.2638713	1.1138856
	0.15		1.3193222	1.0941010
	0.35		1.3658232	0.9803581
		0.1	1.2220998	0.5404720
		0.3	1.2963302	0.6500407
		0.5	1.3907395	0.7532280
		0.7	1.5092073	0.8466337

**Figure 2:**
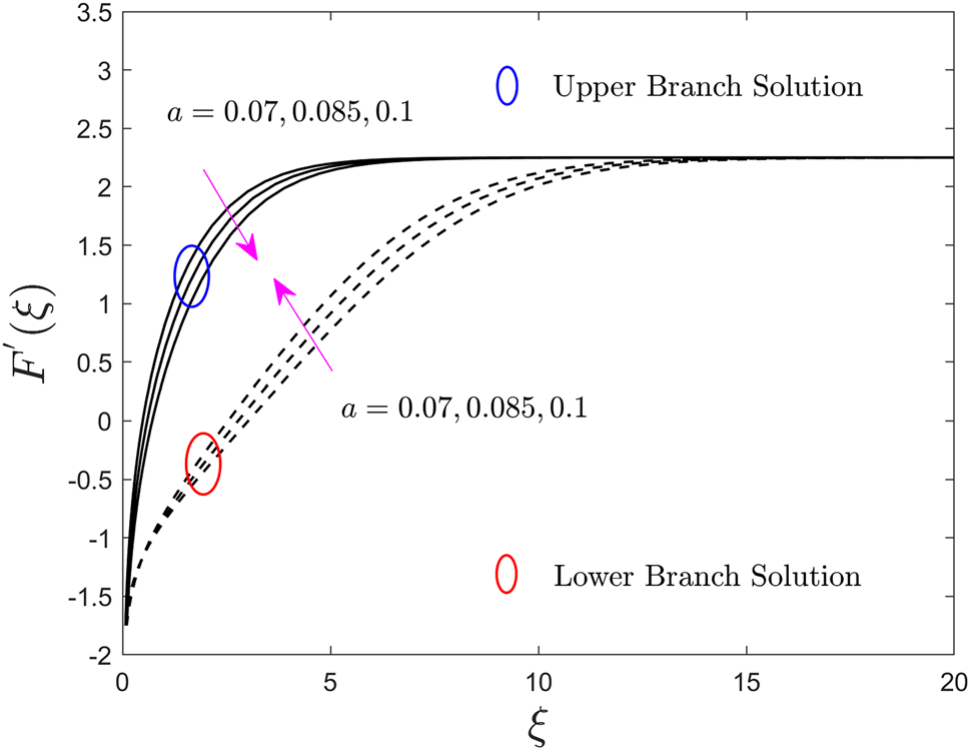
Effect of needle size $a$ on the velocity profiles $F^{\prime}\left( \xi \right)$.

**Figure 3:**
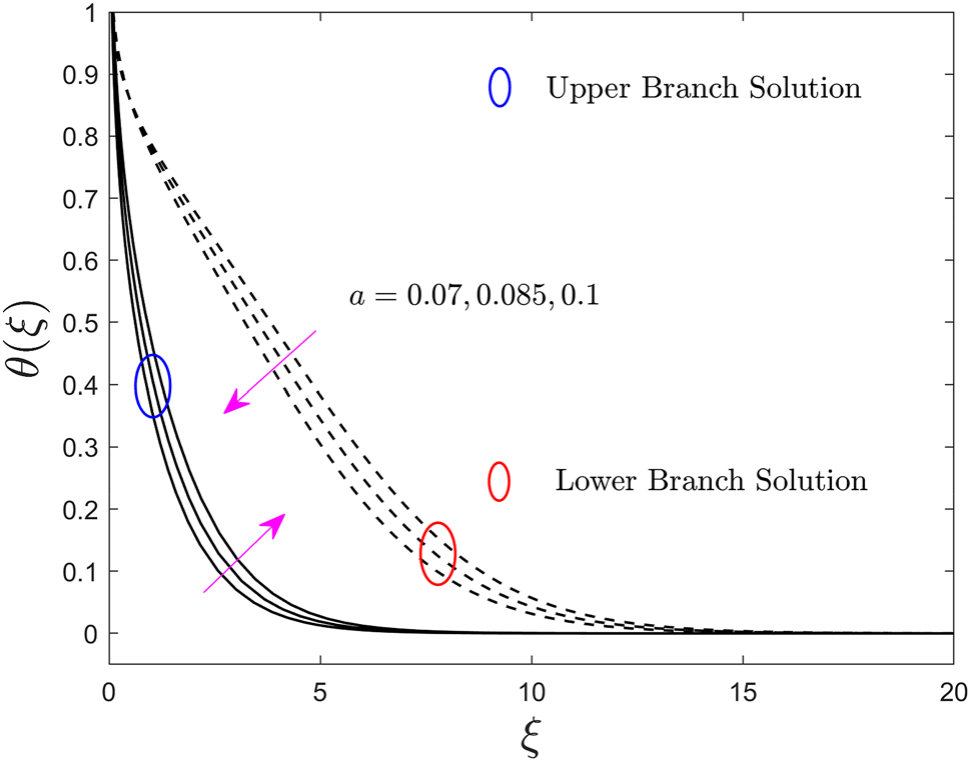
Effect of needle size $a$ on the temperature profiles $\theta \left( \xi \right)$.

**Figure 4:**
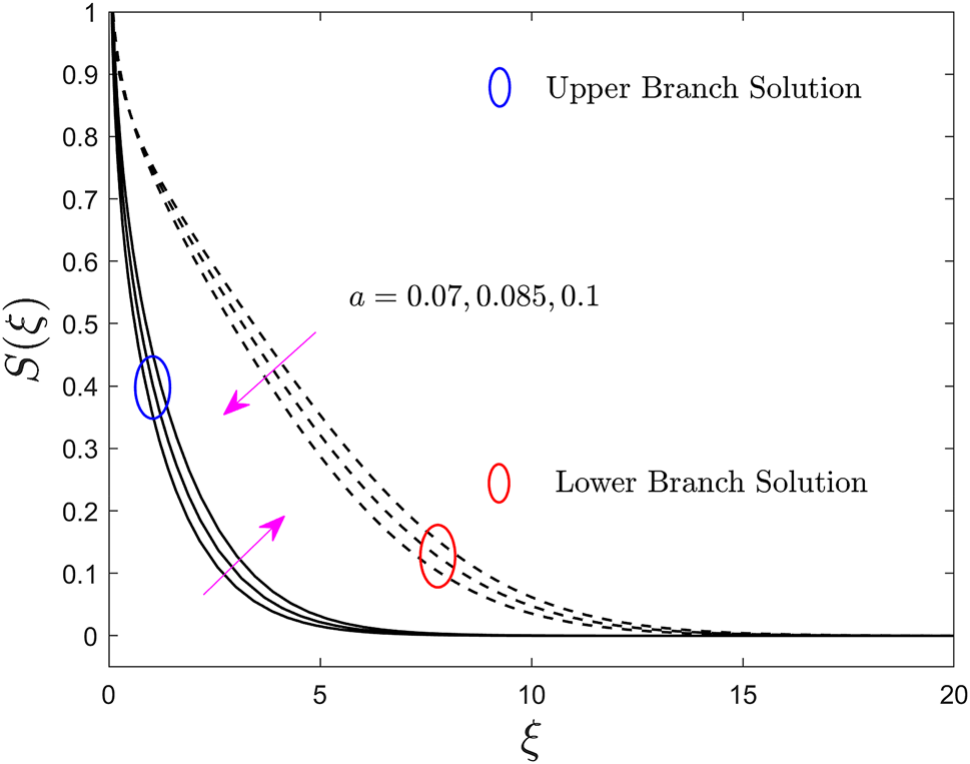
Effect of needle size $a$ on the concentration profiles $S\left( \xi \right)$.

The deviation of the constraint such as the size of the needle *a* on velocity, temperature, and concentration profiles of the (water/AA7072-AA7075) hybrid nanofluid for the UB and LB outcomes are shown in Figs. 2, 3, and 4, respectively. Note from Fig. 2 that growing the size of the needle $a$, results in the shrinkage of $F^{\prime}\left( \xi \right)$ for the UB solution but rises in the LB solution. Generally, the ground surface of a TN in contact with the liquid particles enlarges as the needle's size $a$ increases, increasing the drag force and, as a result, diminishing the velocity field. Moreover, the thickness of the velocity boundary-layer augments because of the improvement in the size of the needle. Alternatively, the temperature and concentration enriches for the UB and declines for the LB results due to the larger value of the parameter $a$. This behavior happens due to the upgrading in the value of the parameter $a$ causes augmentation in the thickness of the TBL and CBL, as a response, the temperature and concentration field upsurges. So, the thickness of the boundary layer can be extra controlled using the constraint $a$. However, the plots further signifying that the gap between the consequences of the UB and the LB is almost similar.

**Figure 5:**
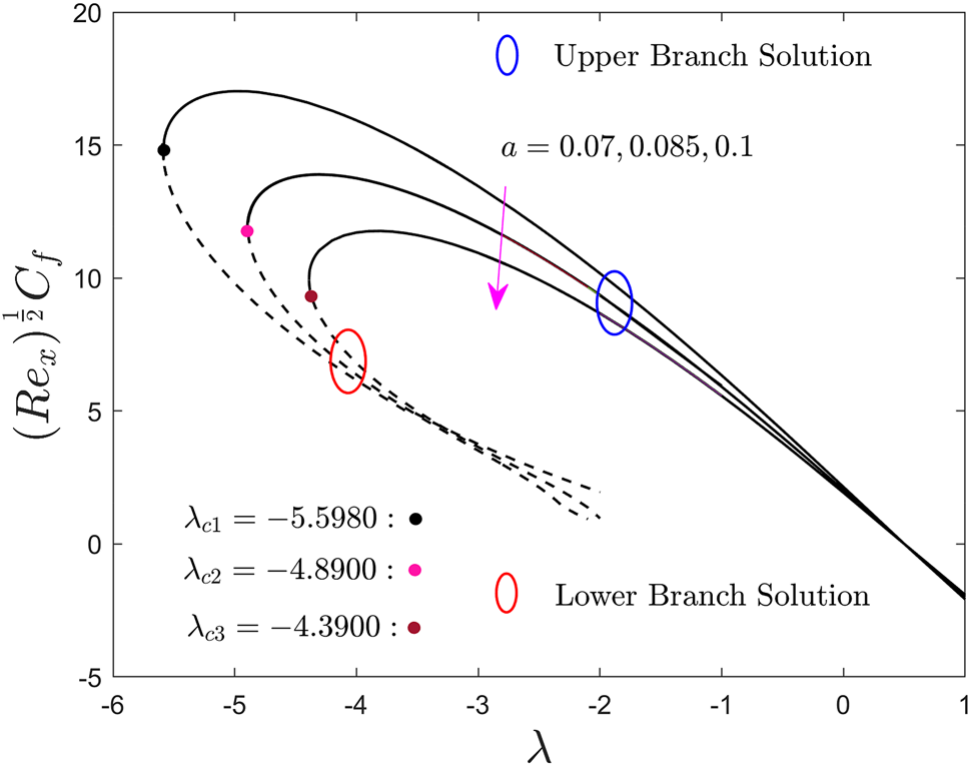
Effect of needle size $a$ on the skin friction coefficient $\left( {{\text{Re}}_{x} } \right)^{\frac{1}{2}} C_{f}$.

**Figure 6:**
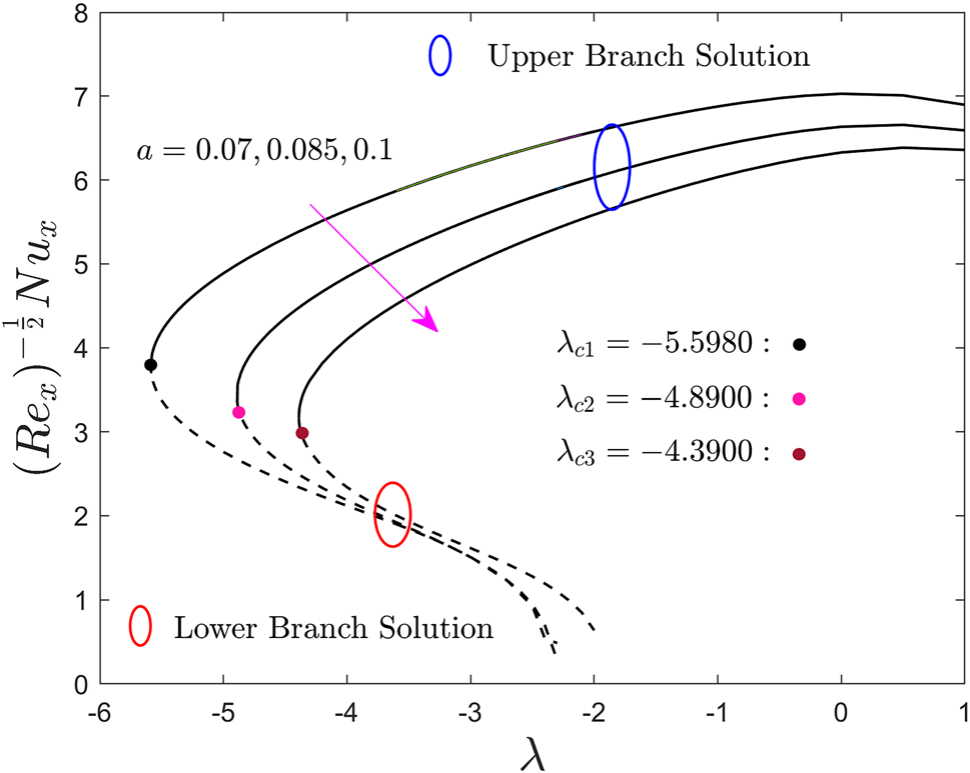
Effect of needle size $a$ on the local Nusselt number $\left( {{\text{Re}}_{x} } \right)^{{ - \frac{1}{2}}} Nu_{x}$.

**Figure 7:**
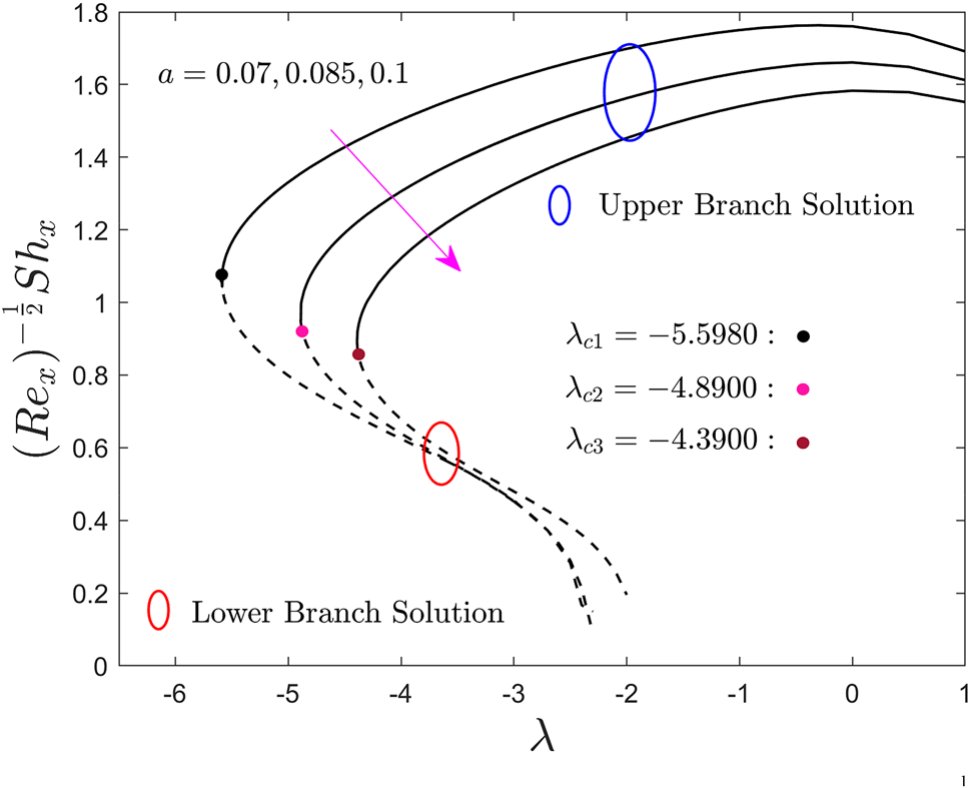
Effect of needle size $a$ on the local Sherwood number $\left( {{\text{Re}}_{x} } \right)^{{ - \frac{1}{2}}} Sh_{x}$.

Figures 5, 6 and 7 are plotted to show the effect of the size of the needle $a$ on the local skin friction factor, the local heat flux, and the local mass flux versus the moving parameter $\lambda$ of hybrid nanofluid for the two different solution branches, respectively. From the graphs it is perceived that, escalating the size of the needle thickness, the local shear stress, heat and mass transfer reduce in the upper branch solution. Meanwhile, a slight increment is seen initially in some specific portion of the range of moving parameter for the LBS and then abruptly reduced due to the larger values of $a$. From Fig. 5, it can be observed that the local shear stress output is completely negative and ultimately goes to zero in the prescribed range ($\lambda > 1/2$). Next, the values of the local shear stress change to positive and keep growing as $a$ upsurges for $\lambda < 1/2$. In general point of view, when the fluid and the thin needle move in the same direction, the negative sign indicates that the solid surface exerts a drag force on the fluid, lowering the local shear stress. Meanwhile, a positive sign of the local shear stress occurs when the TN and the prescribed liquid particles move in the dissimilar direction. At the specific value of $\lambda = 0.5$, the TN and the liquid particles move with same velocity, as a consequence, the local shear stress become zero at the solid surface interface. Moreover, in these plots, the upper and lower solution branches meet at a point called bifurcation, denoted by the distinct small balls such as black, pink, and gray and symbolically represented by $\lambda_{{c_{i} }} \left( {i = 1,2,3} \right)$. As earlier stated, the UB and LB solutions are found for the negative sign of $\lambda$ in which $\lambda > \lambda_{{c_{i} }}$ the result exists, no solution in the range $\lambda < \lambda_{{c_{i} }}$ and unique solution is found for the case of $\lambda = \lambda_{{c_{i} }}$. For each changing values of $a$, the bifurcation points are produced, highlighted by $\lambda_{{c_{1} }}$, $\lambda_{{c_{2} }}$ and $\lambda_{{c_{3} }}$ in all the plots, and their numerical values are also written in the above-mentioned graphs. The strength of the bifurcation values is absolutely decreasing owing to the growing values of the size of the needle, $a$. In this regard, the higher values of the needle thickness parameter suggest that the separation of the BL is accelerated.

**Figure 8:**
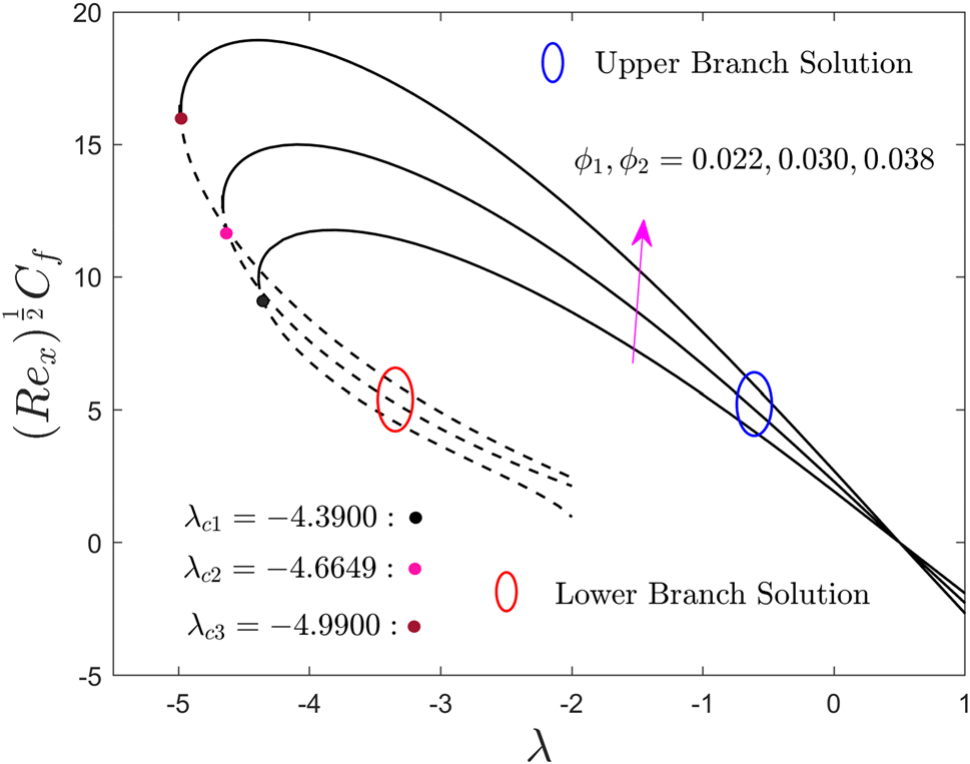
Effect of $\phi_{1}$ and $\phi_{2}$ on the skin friction coefficient $\left( {{\text{Re}}_{x} } \right)^{\frac{1}{2}} C_{f}$.

**Figure 9:**
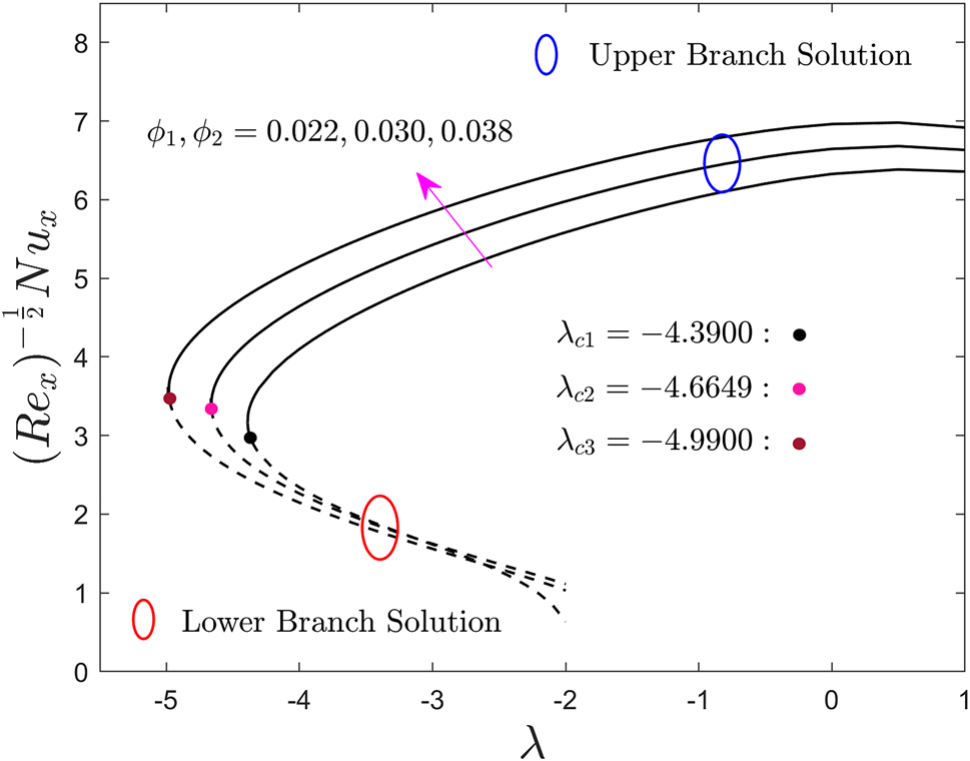
Effect of $\phi_{1}$ and $\phi_{2}$ on the local Nusselt number $\left( {{\text{Re}}_{x} } \right)^{{ - \frac{1}{2}}} Nu_{x}$.

**Figure 10:**
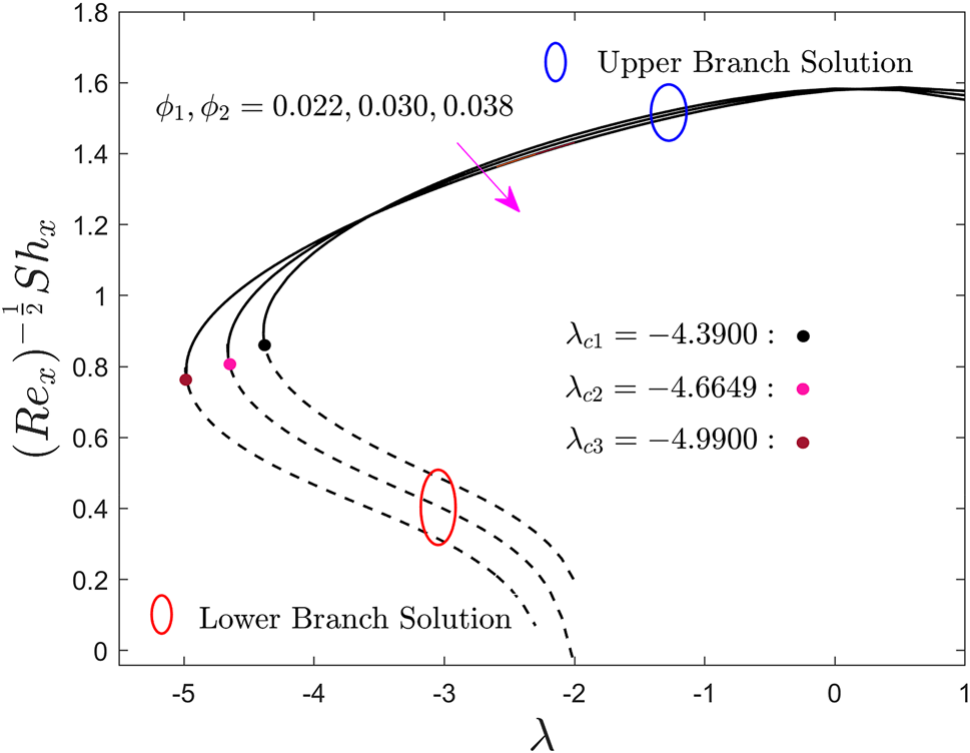
Effect of $\phi_{1}$ and $\phi_{2}$ on the local Sherwood number $\left( {{\text{Re}}_{x} } \right)^{{ - \frac{1}{2}}} Sh_{x}$.

The variations of $\phi_{1}$ and $\phi_{2}$ on the local skin friction factor, the local heat flux, and the local mass flux for the UB as well as the LB solution versus $\lambda$ are shown in Figs. 8, 9, and 10 respectively. Note that, from the products of the UB, the skin friction coefficient first escalates and then declines with the developing values of $\lambda$, while the heat and mass transport rate significantly increase and decrease, respectively. In contrast, the outcomes of the lower branch specify that the shear stress is augmented with larger $\lambda$ while the mass and heat transfer are abruptly decreased. It is understood that the local shear stress has an inverse relation with the flow of fluid such as velocity. So, the improved fractional range of nanoparticles shrinkages the flowability because of the higher amount of quantity it adds. For some vital fact, the fractional upgrade favours the hybrid nanofluid over the pure nanofluid, which has a lesser velcoity field and flows slower, as a result, the local shear stress upgrades. In addition, for larger values of the hybrid alloy nanoparticles, the local shear stress behaviors completely change in the entire region against the moving ratio parameter as compared to the behavior revealed in Fig. 5. Consequently, the first and the second branch of results meet at a bifurcation or critical point and are denoted by $\lambda_{{c_{i} }} \left( {i = 1,2,3} \right)$. The lower branch signifies unstable results and the upper branch is drawn by stable ones. For escalating values of the nanoparticles volume fraction, the values of $\left| {\lambda_{ci} } \right|$ are seen to increase. This trend suggests that adding nanoparticles in the fluid reduces the BL separation. In addition, the gap between the outcomes of shear stress and local heat flux in the upper branch is more as compared to the outcomes of local mass flux but it reverses for the branch of lower solutions.

**Figure 11:**
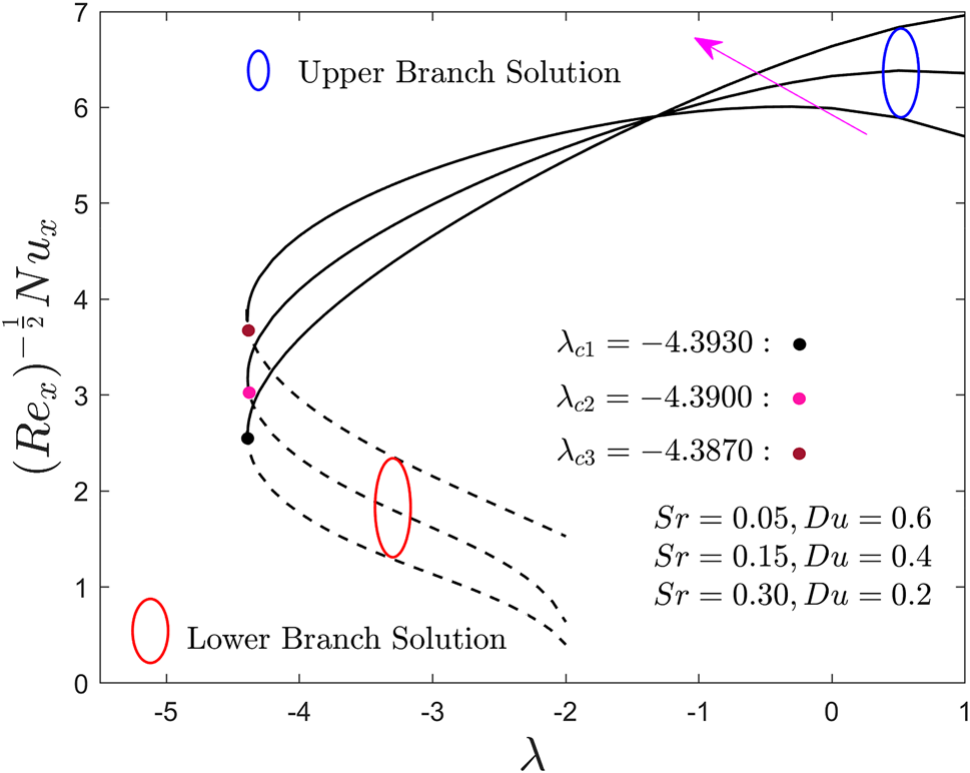
Effect of $Sr$ and $Du$ on the local Nusselt number $\left( {{\text{Re}}_{x} } \right)^{{ - \frac{1}{2}}} Nu_{x}$.

**Figure 12:**
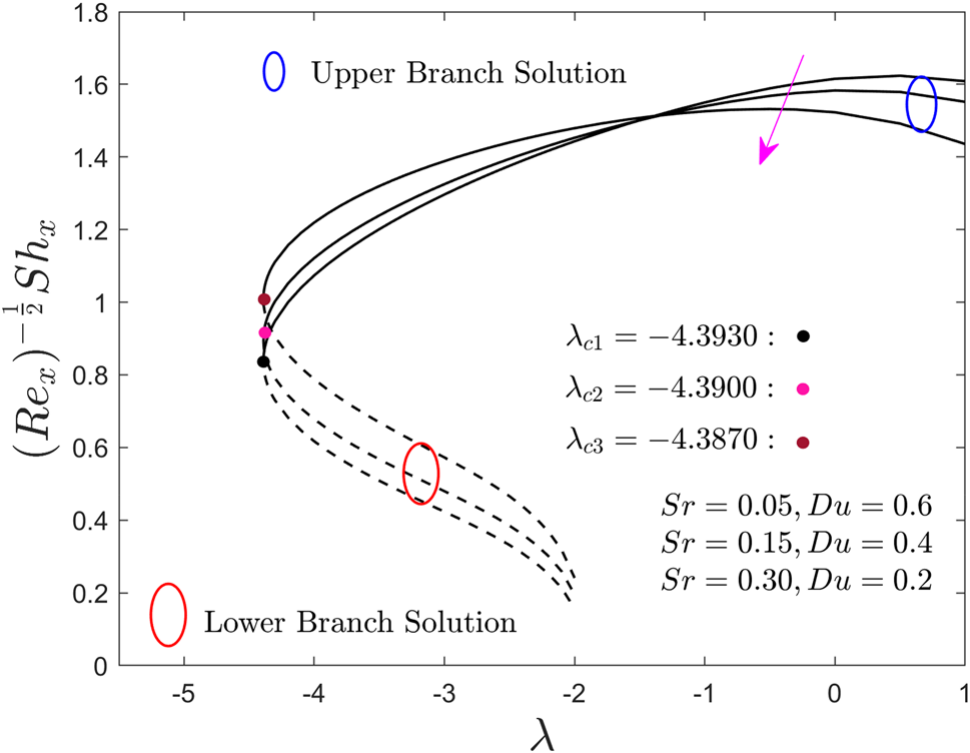
Effect of $Sr$ and $Du$ on the local Sherwood number $\left( {{\text{Re}}_{x} } \right)^{{ - \frac{1}{2}}} Sh_{x}$.

Figures 11 and 12 inspect the influence of Du and Sr numbers on the heat and mass transport rate of the hybrid nanofluid for the lower and upper solution branches against the moving ratio parameter, $\lambda$, respectively. Results reveal that by improving the values of $Sr$ (or lower $Du$), the local heat flux increases and decreases in the upper branch solutions for a specific region of $\lambda$. Meanwhile, the behavior of the local heat flux outcomes is completely diminishing for the LBS. Conversely, the behavior of the local heat mass flux outcomes of the (water/AA7072-AA7075) hybrid nanofluid in both solution branches is opposite (like Fig. 11) due to larger $Sr$ (or lower $Du$) as shown in Fig. 12. From the practical point of view, the impact of the larger $Sr$ (or lower $Du$) is very interesting, therefore, to proceed further it is better to describe that $Du$ and $Sr$ are as the impact of the ratio of concentration difference and the temperature difference and vice versa, respectively. Due to this, the larger diffusive species along the needle surface can improve the local mass transfer flux with $Sr$, while the local heat transfer flux shrinkages with the temperature species and lower $Du$. In addition, it is already mentioned in the previous plots, the small dots in all the figures represent the critical or bifurcation points where the solutions of both branches are the same. This point is denoted by a symbol $\lambda_{{c_{i} }} \left( {i = 1,2,3} \right)$, where $\left( {i = 1,2,3} \right)$ specify the first, second, and third critical points for each change value of Soret and Dufour numbers. Moreover, the critical values are highlighted in the respective figures, which show that by mounting the values of $Sr$ (or lower $Du$) the magnitude of the critical values decrease. This trend shows that by advancement values of $Sr$ (or inferior $Du$) the separation of the boundary layer upsurges.

## Concluding remarks

In the present investigation, the numerical computation of the steady hybrid AA7075 and AA7072 alloys nanoparticles of boundary layer flow are considered towards a thin moving needle under the significant impacts of radiation, Dufour and Soret constraints. The features of the considered distinguished pertinent parameters have been examined with the aid of several figures. The crucial findings of the current paper are revealed as:

- The local heat and mass fluxes enhance owing to decreasing values of Dufor number $Du$ and due to increasing values of Soret number $Sr$ in a specific region of $\lambda$.
- The shear stress, the Sherwood and the Nusselt numbers reduce as the size of needle $a$ increases.
- The shear stress, and the Nusselt number augment in the presence of hybrid nanoparticles $\phi_{1}$ and $\phi_{2}$ while the Sherwood number reduces in a particular region of $\lambda$.
- The two distinct solutions are observed when the TN and free-stream are moving in a different way.
- The velocity declines due to larger thickness of needle $a$ while the temperature and concentration uplift in the branch of first (stable) result. However, the tendency behaves in opposite way in the second (unstable) branch results.

The current work can be extended in various ways, for example, by considering different aspects like mixed convection flow along with motile microorganism or considering different effects like thermal stratification which is very useful in pool type reactor system.

## List of symbols

$$a$$: Needle size
$C$: Concentration of fluid
$C_{f}$: Skin friction coefficient
$D_{B}$: Brownian diffusion coefficient (m^2^ s^−^^1^)
$C_{w}$: Constant surface concentration
$C_{\infty }$: Constant ambient concentration
$c_{p}$: Specific heat at constant pressure (J kg^−^^1^ K^−^^1^)
${\text{c}}_{s}$: Nanoparticle concentration susceptibility
$D_{m}$: Mass diffusivity coefficient (m^2^ s^−^^1^)
$Du$: Dimensionless Dufour number
$F\left( \xi \right)$: Dimensionless velocity of the stream function
$K_{T}$: Ratio of thermal diffusion
$$k$$: Thermal conductivity (W m^−^^1^ K^−^^1^)
$k_{s}$: Mean absorption coefficient (m^−^^1^)
$$Le$$: Lewis number
$Nu_{x}$: Local Nusselt number
$$\Pr$$: Prandtl number
$q_{r}$: Radiative heat flux
$R\left( x \right)$: Shape of the needle
$Sr$: Dimensionless Soret number
$$S\left( \xi \right)$$: Dimensionless concentration
$$R_{d}$$: Radiation parameter
${\text{Re}}_{x}$: Local Reynolds number
$T_{w}$: Constant surface temperature (K)
$$Sh_{x}$$: Sherwood number
$T$: Temperature of the fluid (K)
$T_{\infty }$: Free-stream constant temperature (K)
$U_{w}$: Constant velocity of the moving needle (m s^−^^1^)
$T_{m}$: Fluid mean temperature (K)
$U_{\infty }$: Constant free-stream velocity (m s^−^^1^)
$U = U_{w} + U_{\infty }$: Composite speed or velocity (m s^−^^1^)
$u,v$: Velocity components along $x$ and $r$ axes (m s^−^^1^)
$x,r$: Cartesian cylindrical coordinates (m)

## Greek symbols

$$\alpha$$: Thermal diffusivity (m^2^ s^−^^1^)
$$\theta \left( \xi \right)$$: Dimensionless temperature
$\sigma_{s}$: Stefan-Boltzmann constant (W m^−^^2^ K^−^^4^)
$\lambda$: Velocity ratio parameter
$\mu$: Absolute viscosity (Pa s)
$\xi$: Pseudo-similarity variable
$$\rho$$: Density (kg m^−^^3^)
$\phi$: Solid nanoparticles volume fractions
$$\upsilon_{bf}$$: Kinematic viscosity (m^2^ s^−^^1^)
$$\psi$$: Stream function (m^3^ s^−^^1^

## Abbreviations

2D, 3D: Two and three-dimensional
$$\left\{ \begin{gathered} AA7072 \hfill \\ AA7075 \hfill \\ \end{gathered} \right.$$: Hybrid alloys nanoparticles
Al_2_O_3_: Alumina
BCs: Boundary conditions
bvp4c: Boundary value problem of the fourth-order
CBL: Concentration boundary layer
ICs: Initial conditions
UBS: Upper branch solution
ODEs: Ordinary differential equations
LBS: Lower branch solution
TBL: Thermal boundary layer
PDEs: Partial differential equations
TN: Thin needle

## Subscripts

$$bf$$: Base fluid
$$hnf$$: Hybrid nanofluid
$w$: Wall boundary condition
$$s_{1} ,s_{2}$$: Solid nanoparticles
$\infty$: Far-field condition

## Superscript

*'*: Derivative with respect to $\xi$
